# Supplementary material for: A substrate‐based ontology for human solute carriers
Source: Mol Syst Biol. 2020 Jul 22;16(7):e9652. doi: 10.15252/msb.20209652 (PMC7374931; doi:10.15252/msb.20209652)
Supplement: Supplementary file 1 — Expanded View Figures PDF [file MSB-16-e9652-s001.pdf]

Expanded View Figures

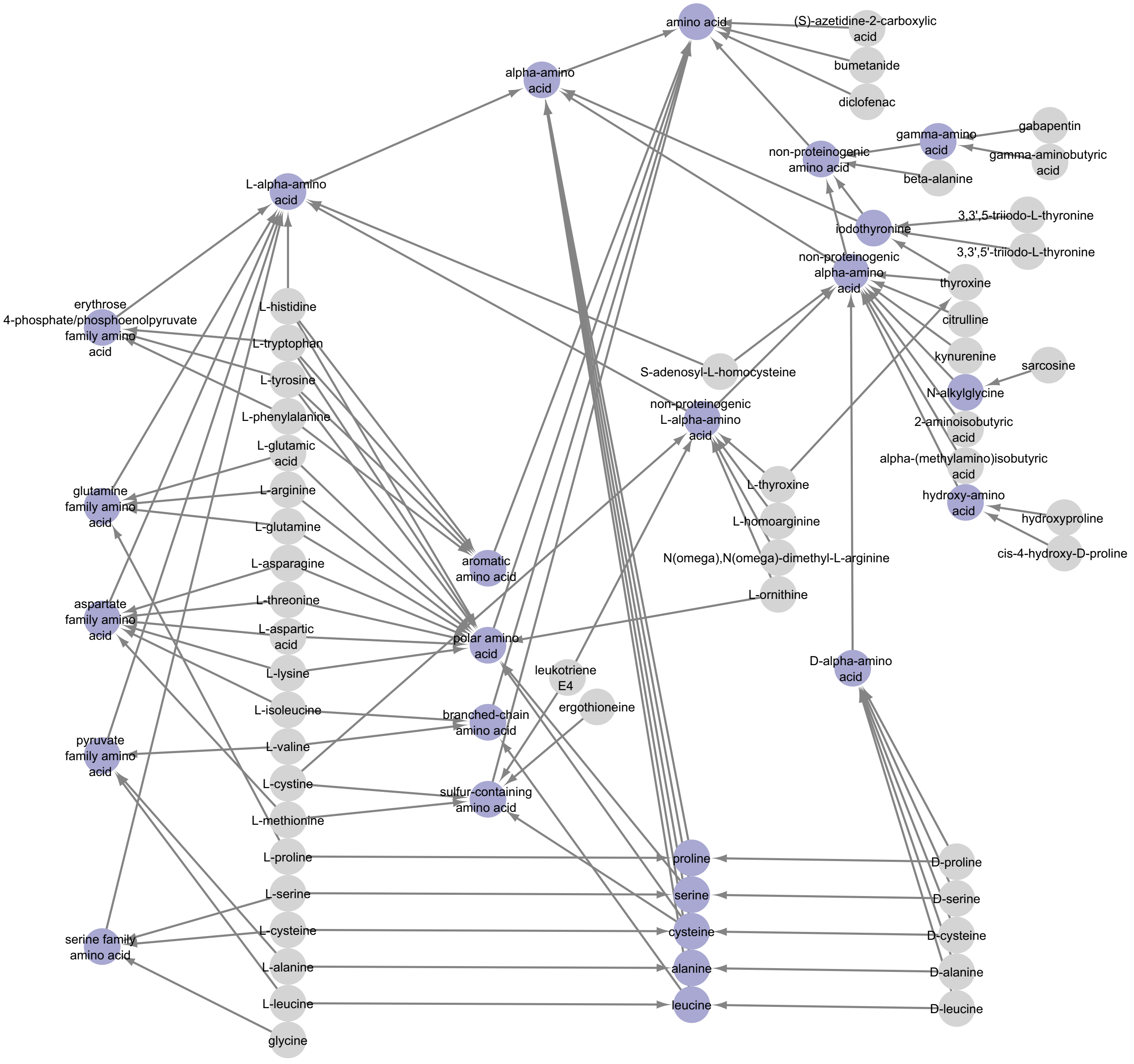

Figure EV1. Exemplified visualization of term “amino acid” and sub-terms.
The proteinogenic amino acids can be found in the lower left area of the network and are grouped metabolically (blue nodes on the left) as well as physicochemically (blue nodes in the right). Please note that this is a sub-graph of the full ontology and SLC substrates (gray) might be connected to more terms in the full ontology.

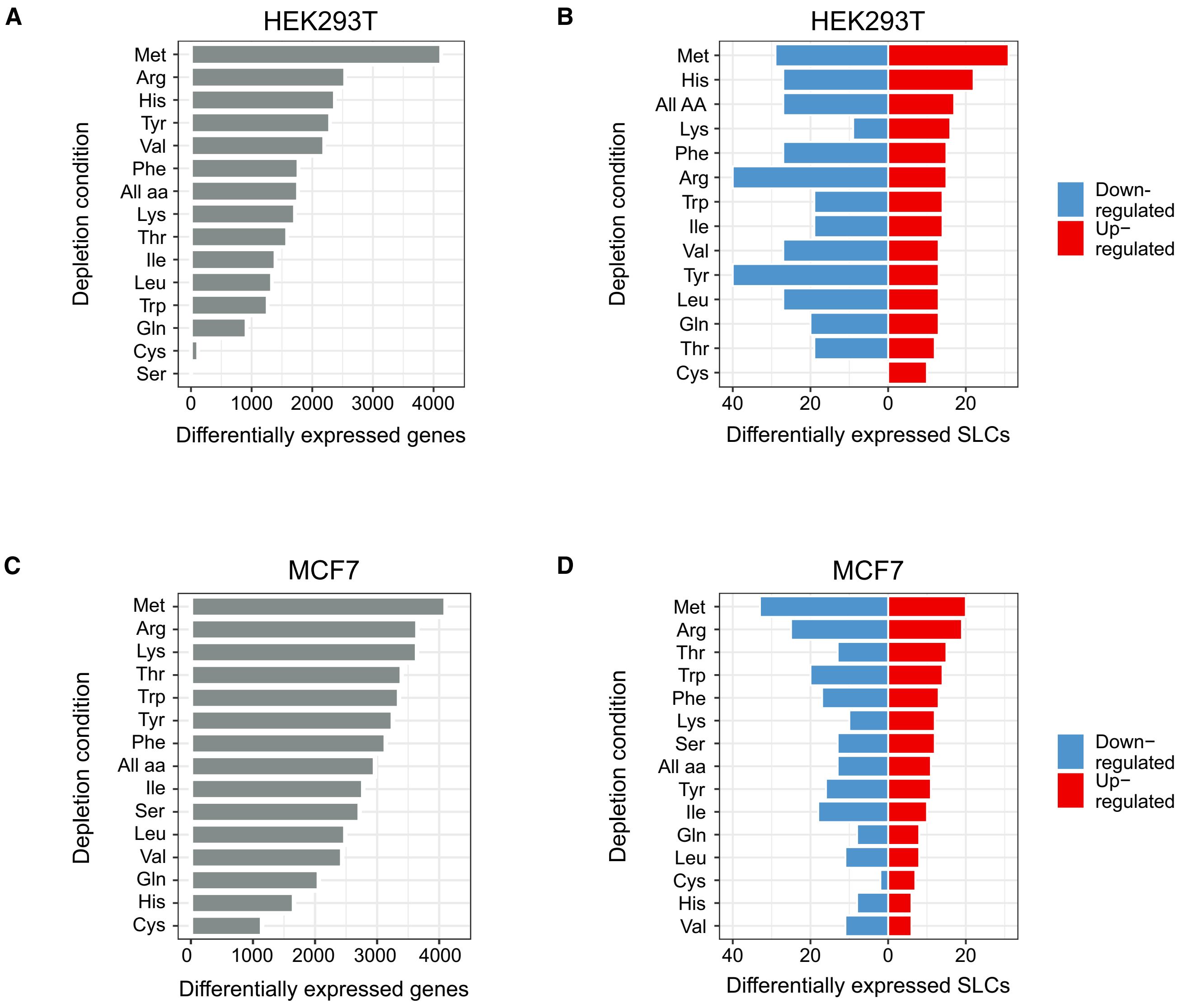

Figure EV2. Upregulation of amino acid transporter gene expression in HEK293T and MCF7 cells after amino acid deprivation conditions.

A, B Number of (A) differentially expressed genes and of (B) up- and downregulated SLC genes for different amino acid depletion conditions in HEK293T cells.

C, D Number of (C) differentially expressed genes and of (D) up- and downregulated SLC genes for different amino acid depletion conditions in MCF7 cells.

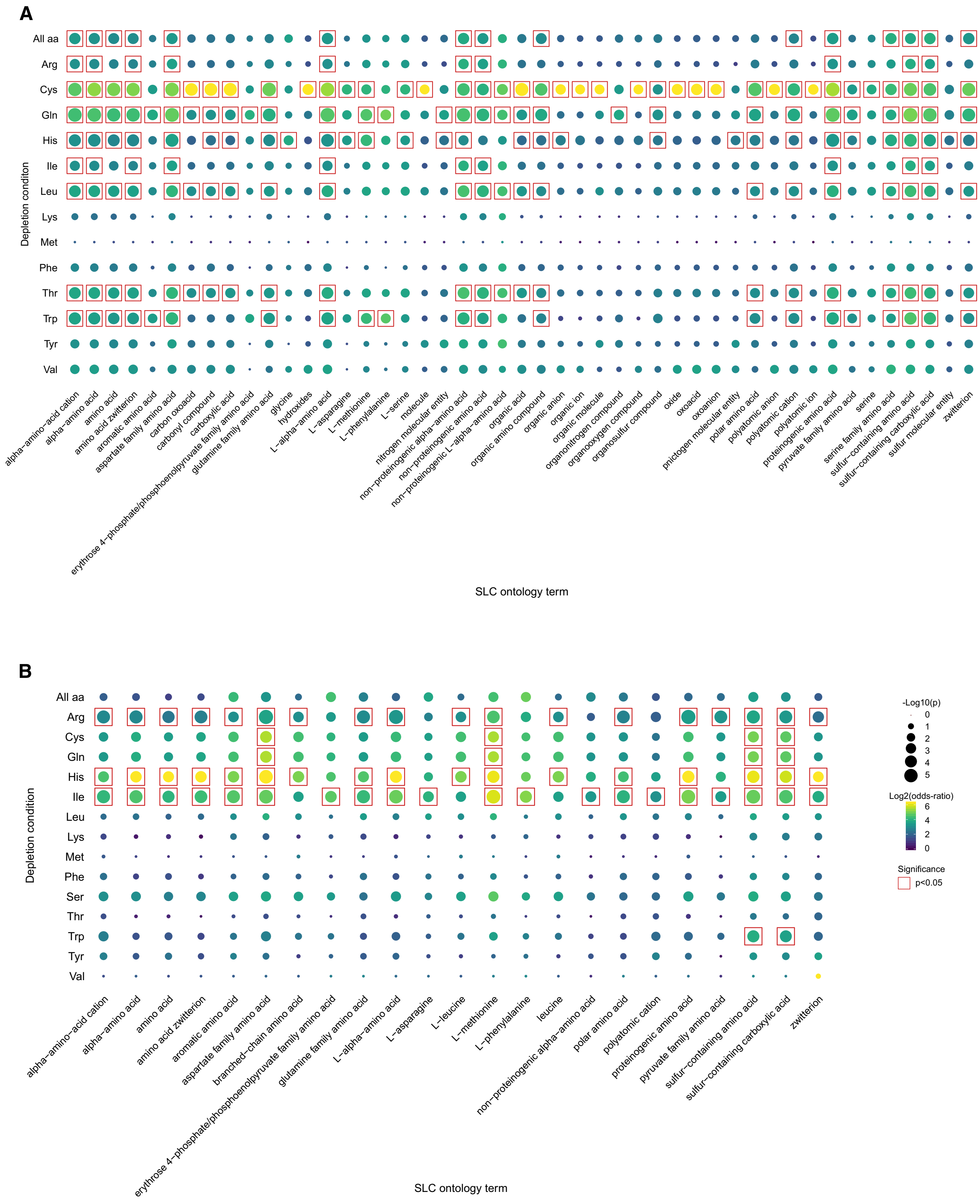

Figure EV3. Unfiltered SLC ontology enrichment results.

A, B SLC ontology term enrichment analysis in upregulated SLCs (all terms) in (A) HEK293T cells and (B) in MCF7 cells. Enrichments were calculated using Fisher's exact test.

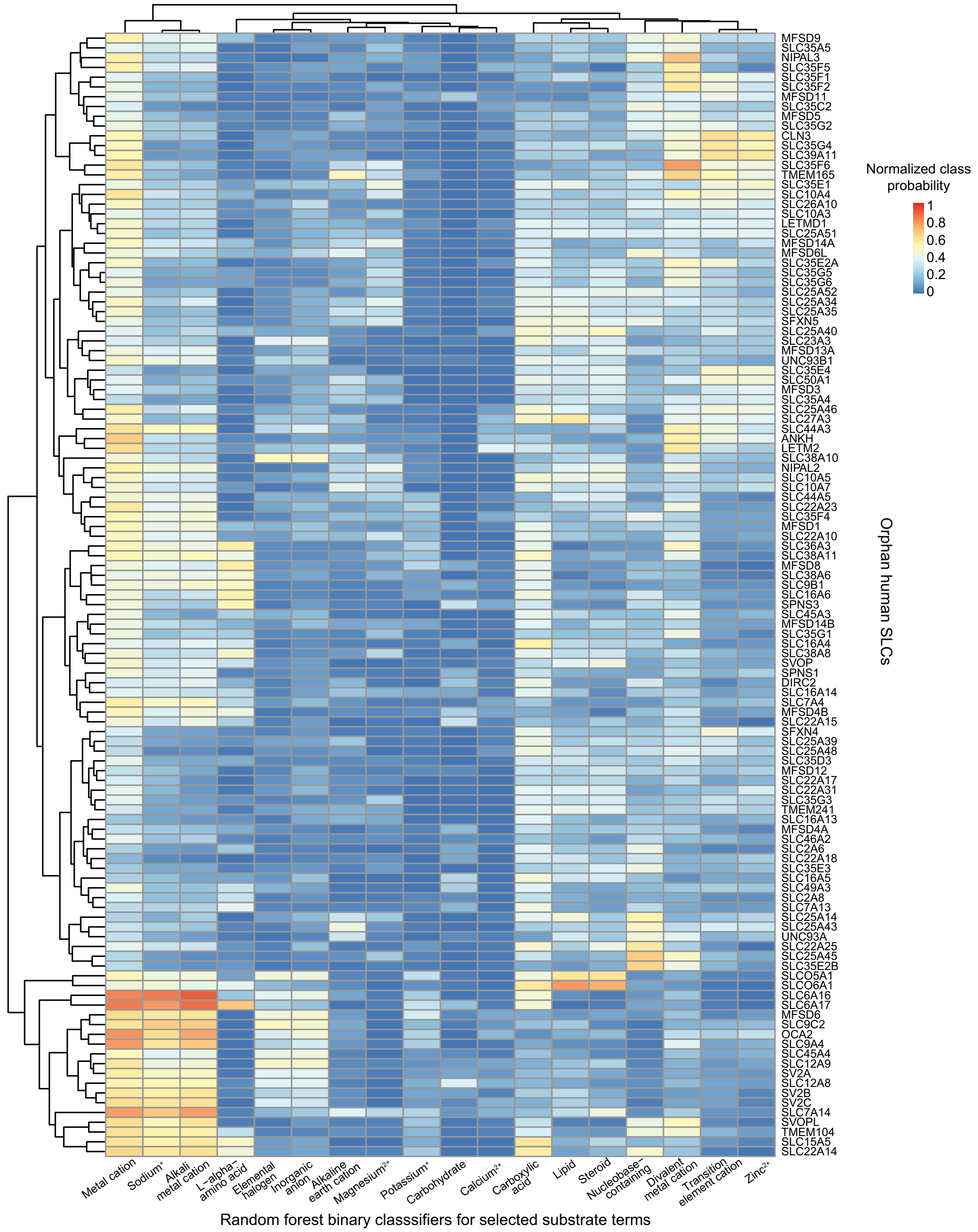

Figure EV4. Substrate probabilities for orphan SLCs.
Probabilities were normalized to a decision threshold of 0.5.
